# Fundamental Lifetime Mechanisms in Routing Protocols for Wireless Sensor Networks: A Survey and Open Issues

**DOI:** 10.3390/s121013508

**Published:** 2012-10-09

**Authors:** Mohammadreza Eslaminejad, Shukor Abd Razak

**Affiliations:** Faculty of Computer Science and Information Systems, Universiti Teknologi Malaysia (UTM), Johor 81310, Malaysia; E-Mail: shukorar@utm.my

**Keywords:** wireless sensor networks (WSNs), network lifetime, energy-efficiency, multi-path, multi-sink, mobile sink, power control, bio-inspired protocols

## Abstract

Wireless sensor networks basically consist of low cost sensor nodes which collect data from environment and relay them to a sink, where they will be subsequently processed. Since wireless nodes are severely power-constrained, the major concern is how to conserve the nodes' energy so that network lifetime can be extended significantly. Employing one static sink can rapidly exhaust the energy of sink neighbors. Furthermore, using a non-optimal single path together with a maximum transmission power level may quickly deplete the energy of individual nodes on the route. This all results in unbalanced energy consumption through the sensor field, and hence a negative effect on the network lifetime. In this paper, we present a comprehensive taxonomy of the various mechanisms applied for increasing the network lifetime. These techniques, whether in the routing or cross-layer area, fall within the following types: multi-sink, mobile sink, multi-path, power control and bio-inspired algorithms, depending on the protocol operation. In this taxonomy, special attention has been devoted to the multi-sink, power control and bio-inspired algorithms, which have not yet received much consideration in the literature. Moreover, each class covers a variety of the state-of-the-art protocols, which should provide ideas for potential future works. Finally, we compare these mechanisms and discuss open research issues.

## 1. Introduction

Wireless sensor networks (WSNs) are composed of a lot of small, low cost sensor nodes that work together to measure various parameters of the environment and send the data to a unique or several sinks where they will be processed [1]. WSNs have a wide range of uses in military, medical, metropolitan and industrial venues. They are employed in many applications such as security surveillance, battlefield and habitat monitoring, intrusion detection, and target tracking purposes. Although reducing the size of sensors could make them cheaper, this also requires that all hardware equipment, specially the batteries, be extremely small. Since the sensor nodes should be functional for a long period of time and battery replacement in harsh environments like battlefields is usually impossible, nodes may lose their energy very fast, thus becoming nonfunctional in a short time. This situation can negatively affect the whole network connectivity, fault tolerance and lifetime. Therefore, optimization for energy consumption is an important issue, especially to prolong network lifetime in WSNs [2]. To address this problem, a variety of approaches are implemented in the area of routing strategies, which play a key role in network functionality and performance [3].

Routing in wireless sensor networks is very challenging. One of the problems that affect the network lifetime refers to nodes in the vicinity of the sink, whose activity imposes a high traffic on this series of sensor nodes. In this state, the nodes that are closer to the sink lose their energy very fast. These nodes are the neighbors located at one hop away from a single static sink. Not only do they utilize energy to relay the data from any other nodes through the network to the sink, but also for sending their own data. This problem is known as the “sink neighborhood problem” [4], which can lead to premature network disconnection. When most of the sink's neighbors' energy is fully depleted, this isolates the sink from the rest of the network, while there is still a huge potential for most of the sensor nodes to continue to perform their tasks and functionalities normally.

One of the basic solutions for the sink neighborhood problem is to employ more than one static sink in the network. Using multiple sinks [5–7] that are statically distributed across the sensor field, it is possible to spread traffic load uniformly among sensor nodes. This can enhance the network lifetime and decrease the end-to-end delays significantly. Another solution for the sink neighborhood problem is to provide some of the network elements with mobile capability [4]. A good strategy to balance energy consumption for data transmission across the network could be replacing the neighbors of the sink. Since nodes' power is limited, a mobilizer unit in mobile nodes consumes the remaining energy faster than under static conditions. The key idea is to maintain the sensors stationary while moving the sink periodically to the parts of the network with sufficient energy. This can prevent network partitioning and consequently prolong the network lifetime. Many protocols [8–11] are proposed for sink mobility, but they differ from each other in the aspect of mobility itself [4]. For instance, in some applications where the sink goes through the network to collect data by itself, an uncontrolled sink movement pattern is applied to the approaches. This means the network may be unable to control the sink movement by applying a specific trajectory based on the nodes' remaining energy or the amount of traffic at each sensor [4]. On the other hand, controlled sink mobility [10,11] can efficiently improve the network lifetime without any negative effects on end-to-end delay.

Although the sink neighborhood problem is one of the most important reasons for network partitioning, there is another problem that can affect the network lifetime. In fact, using a single optimal path [12,13] for every communication may gradually drain the energy of nodes which are located on the route. This causes some problems such as node and link failure due to unbalanced depletion of nodes' batteries across the network. Applying multi-path routing [14,15] in WSNs could result in traffic and energy load balancing over the network. Furthermore, it is not necessary to update the route information periodically, which wastes a remarkable amount of the nodes' power [16].

The sensor nodes are used to forward the data and control packets to the next hop at a maximum power level, which results in fast energy exhaustion. In this state, by employing a power control scheme [17–19] in routing protocols in which the nodes are able to adjust the transmission power level based on the distance from the next hop, the relay nodes can conserve much more energy.

Finally, bio-inspired algorithms [20] have recently been added to the above category as an important class since they can optimize the route construction phase. Bio-inspired protocols which are designed based on insect sensory systems try to construct the shortest path between the source and the destination so that it can conserve much more energy.

Our aim in this paper is to help readers better understand the fundamental energy-aware mechanisms applicable to routing algorithms in wireless sensor networks and point out the potential for improving network lifetime making use of these techniques. We present a comprehensive classification for these mechanisms and discuss a variety of the state-of-the-art energy-efficient routing and cross-layer protocols under this taxonomy. As mentioned before, multi-path methods can avoid network partitioning by distributing traffic loads on most of the sensor nodes while multiple sink and mobile sink methodologies overcome this problem by changing the sink's neighbors periodically and balancing the energy consumption in the sink vicinity. Power control schemes can save nodes' energy by decreasing the power needed to transmit data packets to the next hop in the routing protocols while bio-inspired algorithms can optimize the route construction phase by finding the shortest path for data routing. We categorize the protocols using power control techniques as cross-layer schemes, while the rest are classified as simultaneous mechanisms in the network layer. To the best of our knowledge, our work is the first effort to categorize lifetime improvement strategies applied in routing for WSNs.

The rest of this paper is organized as follows: we present the related work in Section 2. Background and preliminaries is presented in Section 3. In Section 4, we discuss various mechanisms using multiple sinks and mobile sinks to balance the energy consumption of the network. We continue in this section with a presentation of some routing mechanisms using multiple paths, power control and bio-inspired protocols to save node energy and prolong network lifetime. A comparison among different mechanisms is shown in Section 5. Discussion and open issues for future studies is given in Section 6. Finally, Section 7 concludes the paper.

## 2. Related Work

The growing interest in wireless sensor networks on the one hand, and the continual emergence of new architectural techniques in the other hand have inspired some previous efforts for surveying the characteristics, applications and communication protocols for such a technical area [21,22]. In this subsection we point out the features that distinguish our paper and highlight the differences in scope.

The authors in [23] presented full categories of routing protocols for WSNs, as did the authors in [21,24]. However, none of them include the recent energy-efficient mechanisms (such as mobile sink, multi-sink, *etc.*) which could be combined with routing algorithms to increase the network lifetime. Moreover, all of the mentioned approaches only consider the routing algorithms in WSNs from the network structure and the protocol operation point of view. In our paper, we classify not only the routing schemes based on protocol operation, but also from the viewpoint of energy-efficiency.

A taxonomy of different energy-saving strategies applicable in wireless sensor networks is developed in [1] and [25]. According to these surveys, the energy-aware routing protocols in sensor networks are classified by considering several factors such as data cycling, mobility, topology control and data-driven techniques. However, the authors do not focus sufficiently on the network layer and these papers do not include bio-inspired and multi-sink mechanisms for routing protocols. Our survey can serve those who seek deeper insight into energy-efficient routing issues and schemes in wireless sensor networks.

A comprehensive study of mobile techniques for increasing network lifetime is presented in [2]. The authors explained the protocols proposed in all aspects of mobility such as mobile sinks, mobile sensors redeployment, and mobile relays. Although the paper covers a number of routing protocols that support mobility, it does not provide a classification for other energy-efficient techniques applied in routing algorithms. As the best of our knowledge, our paper is the first one that presents a taxonomy of energy-efficient mechanisms, including mobile sink, multi-sink, multi-path, power control and specially bio-inspired schemes, in order to prolong the WSNs' lifetime.

## 3. Background and Preliminaries

### 3.1. Wireless Sensor Network Architecture

**Figure 1.**
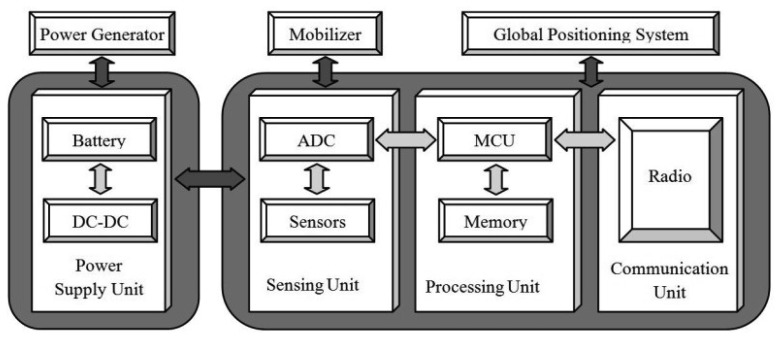
The structure of a typical wireless sensor node [1].

Before describing the high-level taxonomy of energy saving protocols, it is better to have an understanding of the node-level and network architecture for future reference. Figure 1 describes the components of a typical wireless sensor node and their interconnections.

A node consists of four main elements with two optional subsystems as follows:

- A sensing unit, including one or several sensors equipped with analog-to-digital converters for data collection.
- A processing unit, including a microprocessor and memory which cooperate to process the sensed data locally.
- A radio unit used as a transmitter/receiver.
- A power supply unit, including one or more batteries.
- A global positioning system to find the sensors' locations (*optional*).
- A mobilizer unit to change their position (*optional*).

It is worth mentioning that as indicated, the last two components are optional and may be used based on application requirements [1].

### 3.2. Sources of Energy Consumption in WSNs

Power failure in WSNs depends on the nodes' characteristics. For example, Raghunathan *et al.* [26] have shown that the power properties of a Stargate sensor node are different from those called motes. However, they do share the following common points:

- The energy consumption of communication unit is much higher than that of the processing unit. For instance, the energy needed for executing 3,000 instructions in a CPU is equal to the energy needed for transmitting just 1 bit of data [27], so a tradeoff between computation and communication is necessary.
- The radio unit consumes energy at the same level in reception mode, transmission mode and idle state. In order to save energy, it is better to turn off radio whenever it is not in used.
- The sensing unit can be a main source of power consumption depending on the application in use, so an appropriate policy should reduce the energy utilization in this unit significantly [1].

According to the above architecture and power failure issues, the routing protocols are classified into three main categories based on the network structure, namely *flat*, *hierarchical*, and *geographic algorithms*. At the next subsection, this general classification will be discussed.

### 3.3. General Classification of Routing Protocols in WSNs

Is mentioned, according to the network structure, routing protocols in WSNs can be divided into three categories [21]: data-centric (flat), hierarchical, and geographic (location-based). They are described as follows:

- *Data-Centric protocols:* Multi-hop data-centric routing protocols are basically the first class to be introduced in WSNs. Considering a large number of nodes in sensor networks, flat algorithms employ *query-based* mechanisms in which the sink node only requests the desired data in order to prevent continuous data transmissions and thus save power. In this group, Sensor Protocols for Information via Negotiation (*SPIN*) [28], Directed Diffusion [29], Energy-Aware Routing (*EAR*) [30], Rumor Routing [31] and Minimum Cost Forwarding Algorithm (*MCFA*) [32] are some of the most famous flat algorithm paradigms.
- *Hierarchical protocols:* Different from the flat category, in hierarchical protocols that utilize a clustering scheme, nodes are assigned different roles or functionality. In fact, energy conservation can be achieved in these protocols by some aggregation and reduction of data in so-called cluster heads (CHs). In this class, Two Tier Data Dissemination (*TTDD*) [33], Low-Energy Adaptive Clustering Hierarchy (*LEACH*) [34], Threshold-Sensitive Energy-Efficient Sensor Network Protocol (*TEEN*) [35], Adaptive Periodic Threshold-Sensitive Energy-Efficient Sensor Network Protocol (*APTEEN*) [36] and Power-Efficient Gathering in Sensor Information Systems (*PEGASIS*) [37] are some inspiring protocols.
- *Location-Based protocols*: The possibility to apply position information in routing schemes will be used in location-based algorithms to route data towards the desired regions in the sensor field. This can save energy by limiting the flooding through the network [22]. *GPSR* [38], *GAF* [12], and *GEAR* [13] fall in this class.

## 4. Lifetime Improvement Mechanisms in Routing

**Figure 2.**
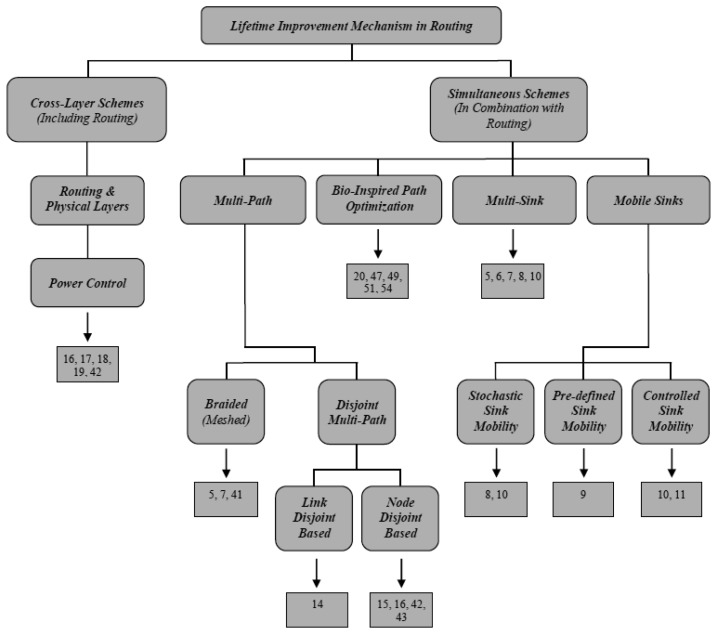
Classification of fundamental lifetime improvement mechanisms in routing protocols for WSNs.

In the next subsections, the main categories of energy-aware mechanisms applied to routing protocols in WSNs will be discussed in detail. Figure 2 shows the taxonomy of the methods covered in this paper.

In this figure, the numbers represent the corresponding references. However, some protocols [5,7,8,10,16] fall in more than one category. Lifetime improvement mechanisms in routing protocols for WSNs are basically divided into two main categories: *simultaneous schemes* and *cross-layer schemes*. Simultaneous schemes [21] usually refer to the mechanisms which could be combined with routing algorithms in order to achieve a specific goal like energy-efficiency. In WSNs, these mechanisms are classified based on the protocol operation. However, cross-layer schemes [1] investigate different layers simultaneously to make the protocol more energy-efficient. In the following, we discuss the various classes under these two categories.

### 4.1. Multi-Sink Mechanisms

As mentioned before, network partitioning caused by energy depletion around the sink (the sink neighborhood problem) is one of the main issues that affect the network lifetime. Therefore, many techniques have been used in previous works to overcome this problem. One possible method is to employ multiple sink nodes throughout the network. Researchers who work on multi-sink mechanisms believe that by increasing the number of static sink nodes one can distribute the traffic load all over the network and consequently balance energy consumption around the sink. Finding an optimal location for the sink nodes and looking for low cost paths from each source node to one or several sinks [5] are the main concerns in this research area.

***Multi-Sink Directed Diffusion (MSDD)***, which was proposed in [5], is a kind of multi-sink approach that employs the basic idea of a Directed Diffusion (DD) routing protocol to construct routes from each source node to the nearest sink node. Network lifetime could be increased in this protocol by switching the data flow to the next nearest sink when the power level of relay nodes on the primary path falls below a certain threshold. Just like the DD algorithm, the sinks propagate interest messages through the network to find the sources which contain the data of interest. When a source node receives such messages from multiple sinks, it responds by broadcasting an exploratory data (ED) message through the network. Then bi-directional paths are constructed towards the source node and the sinks start to send positive reinforcement messages to the source. In this state, if the source node accepts all reinforcement messages from multiple sinks, the data packets should be forwarded to all of them, which imposes a large overhead caused by the redundant data. Therefore, it registers the neighbor node that sends the positive reinforcement with smallest *Hop_Count* value into the *Path_List* table. It also retains the information about other paths to use them as backup routes when the residual energy of the primary path falls below a certain threshold.

**Figure 3.**
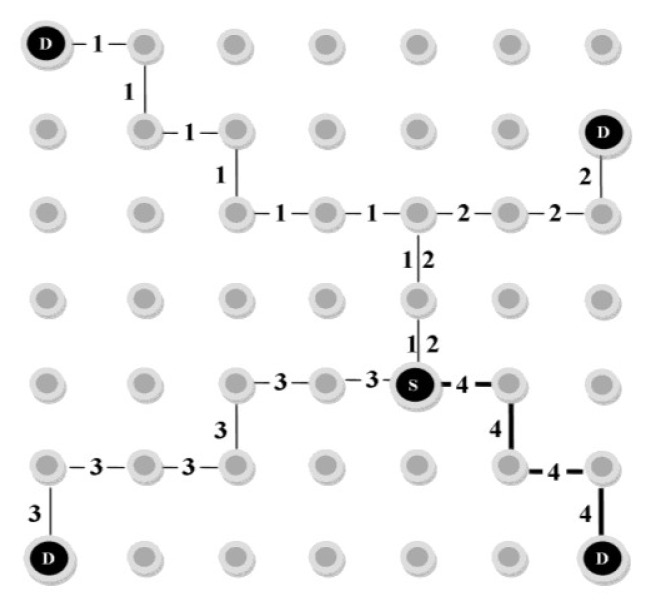
Path selection with minimum hop count [5].

In some situations, as shown in Figure 3, a single neighbor of the source may be shared among several paths from different sinks. Thus, by choosing this node, data packets will be relayed towards all the paths including this neighbor. In order to avoid this problem, each sink node assigns a random number as *Path_Id* to the positive reinforcement messages. These path identifiers that distinguish the paths from each other are also registered in Path_List table. As illustrated in Figure 3, D represents the sink node (destination) and S indicates the source. There is also a source neighbor that is common between the paths with Path_Ids 1 and 2. In MSDD, a negative reinforcement message is employed to inform the source node of a path failure. In this state, it then removes this path from its Path_List table.

Simulation results [5] show that MSDD could enhance the average energy of network nodes and the energy of nodes with the minimum energy by increasing the number of sink nodes. The authors also proved that connection lifetimes up to three times longer could be achieved using a multi-path routing algorithm. The routing overhead of Directed Diffusion is decreased in MSDD, which results in up to two times higher network lifetime. Nevertheless, the algorithm could only be used in query-driven applications according to the main operation of Directed Diffusion family protocols.

***Gradient-Based Routing Protocol for Load Balancing (GLOBAL)*** [6] is another multi-sink protocol that maximizes network lifetime with the help of a new gradient model. This algorithm selects the least-loaded path for data forwarding that also excludes the most overloaded sensor nodes. By applying this method, network lifetime is not limited by the short lifetime of such overloaded nodes. Each sensor node in this protocol computes its residual energy depletion rate (*REDR*) that will be used later in gradient field construction phase. Equation (1) shows the *REDR* for node *i* where: *α* is the weighting factor, *REDR_old_* indicates the previous *REDR*'s value for this node and *REDR_sample_* represents *REDR* during past *T* seconds:

(1) $${\text{REDR}}_{i}=\alpha \times {\text{REDR}}_{\text{old}}+(1-\alpha )\times {\text{REDR}}_{\text{sample}}$$

*REDR_sample_* is calculated by the Equation (2) as follows:

(2) $$\text{REDRsample}=(1-\frac{\text{Current Residual Energy}}{\text{Current Residual Energy}-T})/T$$

The protocol consists of two phases as follows: (1) *Gradient field construction and data forwarding phase:* in this stage, an advertisement (ADV) message is flooded by each sink but not at the same interval to ensure that there is no interference between two consecutive floodings. It contains the three following fields: (a) *hcnt:* the number of hops from the sink, (b) *sum-redr*: the sum of nodes' *REDR* on the path and (c) *max-redr*: the maximum *REDR* value of nodes on the path. When the source node *i* receives an ADV message for the first time, it assumes that the acquired path is the shortest one and uses it for data transmission. Then it computes its gradient *G_i_* according to Equation (3), saves it in memory, updates ADV message and finally rebroadcasts it through the network:

(3) $$G_{i}=\beta \times \mathrm{sum}-{\text{redr}}_{L}+(1-\beta )\times \max-{\text{redr}}_{L}$$

In this equation, *sum-redr_L_* = the path's *REDR* + node *i*'s *REDR*, *max-redr_L_* = the maximum *REDR* on the path including node *i* and *β* is a weighting factor of these parameters. If node *i* experiences a lower loaded path than the first one so that its length does not exceed a specific number of hops and its gradient is lower than *G_i_*, it replaces this newly discovered path with the previous one. (2) *Gradient field maintenance:* during the network functionality, the gradient field should be refreshed. Instead of flooding, GLOBAL updates this field during data transmission by exploring overhearing packets from other neighbors. This can reduce overhead throughout the network.

Simulation results [6] indicate that GLOBAL improves the network lifetime by 50% and 18% more than shortest path routing (SPR) and CPL, which is a gradient-based routing using the cumulative path load only, respectively. The philosophy behind this improvement is that in GLOBAL, the traffic load of the most overloaded sensor over the path and a weighted average of the cumulative path load are used by an independent node to determine its gradient. The main drawback of GLOBAL is the high control overhead caused by sinks' advertisement flooding in the gradient field construction phase.

The authors of the ***Multi-Sink and Load-Balance Routing Algorithm (MSLBR)*** [7] proposed a method to prolong the network lifetime through distribution of loads among sink neighbors that are called deputies. In this way, individual data packets generated by each source node can choose different deputies randomly as their destination and traverse different paths to reach them. Moreover, the source node uses a forward factor based on dividing the residual energy of its neighbors by their shortest hops to the destination. The quotient of this division used to find the next hop during a routing phase. MSLBR has three phases as follows: (1) *Network topology discovery:* In this stage, all nodes use a beaconing mechanism to broadcast their deputy table periodically. As shown in Equation (4) the beacon of node *N_i_* is a set of four items:

(4) $$\text{Beacon}=\{N_{i},E_{i},D_{j},H_{i,j}\}$$

where *E_i_* = the energy level of node *N_i_*, *D_j_*, = the identity of a deputy and *H_i,j_* = minimum hops between node *N_i_* and deputy *D_j_*. All of the entries in the deputy table of node *Ni* should be broadcasted periodically in a round-robin manner. (2) *Routing table updating:* all sink nodes start to broadcast beacons at the initialization phase of a network. After that, the deputies that receive these beacons from the relative sink node rebroadcast them to the other neighboring nodes in the network. When the beacon is received by node *N_i_*, it firstly updates its neighbor table and deputy table. After that, it begins to broadcast the beaconing message periodically. (3) *Packet transferring:* it is the last phase of routing stage. When the node *N_i_* completes the updating process by refreshing the neighbor and deputy tables, it is able to generate and forward data packets to deputy nodes if it finds more than one valid entry at the mentioned tables.

In order to balance the loads among all deputies, two methods are used in MSLBR: (a) in a round-robin mechanism, the destination of a new packet can be set to one of all deputy nodes in the network; (b) according to the forward factor of neighbors, a node can relay the packets to the next hop. Since the kernel of MSLBR protocol is to route a packet to a deputy randomly, it can efficiently distribute the traffic load on most of sensor nodes and extend the network lifetime [7].

The test-bed experiments [7] show that MSLBR protocol has on average 7.1% and 14.4% longer lifetime compared to the primary based routing (PBR) and energy level based routing (ELBR) algorithms. However, the time cost for updating routing table and finding the match deputy for a packet in MSLBR is a bit high. Therefore, the data transmission delay is increased rather more than in the two other approaches.

### 4.2. Mobile Sink Mechanism

Recently another solution for network partitioning caused by energy depletion around the sink has been by applying an efficient mobile sink strategy into the multi-hop routing protocols. Since the sinks' neighbors should forward their sensed data accompanied with data packets from nodes that are far away, they lose their energy faster than the other parts of the network. Instead of replacing these nodes, the key idea is that of maintaining the sensors stationary while moving the sink periodically to the parts of the network with sufficient energy. Since the sink's neighbors are permanently changed in time, the energy consumption and the traffic load could be balanced all over the network. On the other hand, employing only one mobile sink can avoid disconnection between the sensor nodes and the sink that caused by sink neighborhood problem. It consequently increases the network lifetime [8].

Three basic types of sink mobility patterns are *Random/stochastic mobility*, *controlled mobility*, and *fixed path/predictable mobility*. In stochastic mobility, a random path is followed by the sink node in the network. Meanwhile, the data collection from the sensor nodes will be implemented based on a pull strategy where the sink requests the data from either one or *k* hop neighbors. According to the controlled mobility pattern, the sink can move through the network autonomously and change its position based on the energy factors in the sensor field. In the predictable mobility method, on the other hand, the sink node moves along a fixed path [39].

#### 4.2.1. Random/Stochastic Sink Mobility

Chatzigiannakis *et al.* [8] presented three randomized patterns for sink mobility accompanied by two data collection mechanisms. They showed that randomized methods can prolong the network lifetime, at the expense of increasing data latency. These schemes are as follows: (1) *Random Walk and Passive Data Collection:* in this method the protocol uses two following techniques: firstly, it chooses a random path in the sensor field so that the next step for the sink movement is achieved by a stochastic function called *M_random_*. This function produces two random uniform values. The first one is an angle in [-π,π] radians that determines the deviation from the current direction of mobile sinks. The second one indicates a distance *d* ∈ (0, d_max_] that the sink node should travel along the defined direction. Secondly, data collection from the source nodes is implemented by a (passive manner) pull strategy. It means the sink asks one hop or *k*-hop neighbors to send their information (where, *k* > 1). (2) *Partial Random Walk with Limited Multi-hop Data Propagation:* The network in this method is a square of size *D* × *D* and *R* is the fixed radio transmission range of the nodes. All of the sensor nodes form a graph *G_o_* during the network initialization. This graph partitions the network in *i* × *i* square regions. The mobile sink is located on one of the nodes of graph *G_o_* at the beginning. Then a random function *M_graph_* computes the next place for the sink repositioning among the neighbors of current vertex by selecting one of them uniformly randomly. If $i=\lceil D\times \sqrt{2}/R\rceil$, the sink can cover all of the sensor nodes in *D/i* × *D/i* area when it locates at the center point of each square. Data collection protocol is a tree with the sink at root. The sink periodically broadcasts a beacon including hop count and TTL fields. When a sensor node receives a beacon, it updates these fields and specifies its parent in this tree. After that, it starts to send data to the mobile sink.

In comparison to the first method, the distance traveled by the sink node is reduced efficiently and the sensor nodes are covered much faster at the expense of computational and communication overhead. (3) *Biased Random Walk with Passive Data Collection:* in biased random mechanism, the sink node collects some information about the areas that were visited and then decides to change its position based on this information. If the sink is currently on vertex *u*, the probability of visiting a neighboring vertex *v* are *p*(*f*)*_v_* and *p*(*d*)*_v_* based on frequency of visiting this area and the node density in this place respectively. Therefore, the final probability for sink repositioning to the area related to vertex *v* is presented in Equation (5), where *α* and *β* are positive weighted factors so that *α* + *β* = 1:

(5) $$p_{v}=\alpha \times p{\left(f\right)}_{v}+\beta \times p{\left(d\right)}_{v}$$

A passive data collection is also used for this protocol, but in comparison with the first method, it has lower latency and higher delivery ratio. Nevertheless, the main drawback of the random sink mobility is that data collection from all of the sensor nodes through the network is not guaranteed [8].

The simulation results [8] show that the random sink mobility could apply in delay tolerant applications as well. It seems the delivery delay would be decreased by trading off some energy efficiency. However, the results are not affected by topology settings. The main drawback of random waypoint algorithms is that the sink cannot visit all of the source nodes in the field. On the other hand, the delivery ratio depends strictly on the speed of the sink node. In this protocol, when s = 8 m/s the success rate is about 95%. This metric will be reduced to 85% when the sink node decreases its speed to s = 4 m/s. Increasing the sink speed may cause more energy wastage across the network. In this state, the nodes which are one hop away from the sink node do not have enough time to relay all packets to the sink and this results in some packet losses and data re-transmissions. In comparison with Directed Diffusion, the algorithm achieves 40% lower energy consumption.

#### 4.2.2. Fixed Path/Predictable Sink Mobility

Jean and Hubaux [9] proposed a protocol based on fixed path/predictable sink mobility and joint routing in order to enhance the network lifetime. They realized that the maximum lifetime for the wireless sensor network could only be achieved if the sink path covers the periphery of the sensor field. The algorithm uses two patterns for the sink movement while the sensor nodes are distributed in a circular-shape network. The first one is a discrete mobility strategy in which a sink can move and stop periodically. If the protocol employs a time synchronization method among the sensor nodes accompanied with a time scheduling for discrete trajectory, each sensor node knows the position of the sink at a given time and consequently can select the best path for data transmission. The other one is a continuous mobility pattern with the help of location information. In this method, the network topology may change due to the mobility, so the sink node should broadcast a data query time by time in order to update the routing table of the nodes. An online route construction strategy is possible if the sensor nodes and the sink are aware of their location and the sink node announces its position to all nodes.

**Figure 4.**
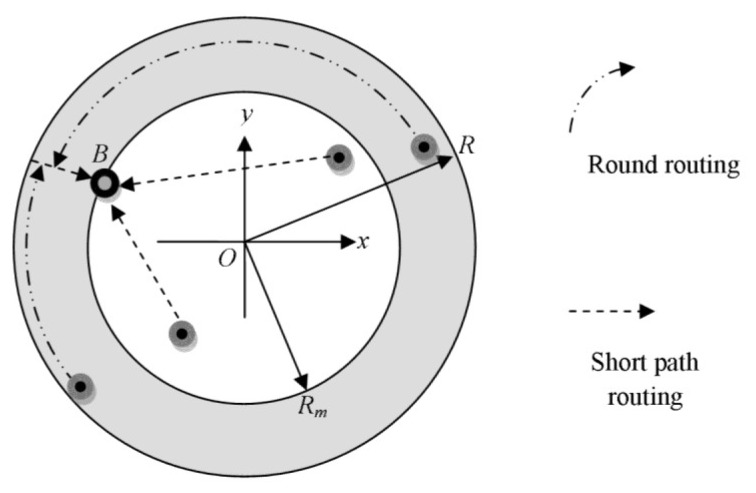
Joint routing mechanism and sink mobility [9].

Joint routing is shown in Figure 4, where the network is partitioned into two parts: a circle of radius *R_m_* < *R* and the annulus-shape area between the periphery of the network and the mentioned circle. The sink trajectory is only the circle of radius *R_m_*. There are two kinds of routings towards the mobile sink as follows: (1) the sensor nodes that located within the inner circle should transmit the data on shortest path; (2) the packets sent by the other nodes within the annulus area take a round routing around the center *O* to reach *OB* position. After that, they will be forwarded to the mobile sink through a short path. The direction of round routing should be in such a way that the overall traversed distance will be minimized. The philosophy behind the joint strategy is that through reducing the radius of the network from *R* to *R_m_*, it can limit the area that employs short path routing and hence achieve a lower network load.

Although the joint routing mechanism is extremely dependent on the shape of the sensor field, the experimental results [9] show that in comparison with static base station, it can enhance the network lifetime more than 500% by exploiting the potential energy capacity all over the network. However, the packets produced by the nodes in an annulus-shaped area may experience more end-to-end delays due to the circular routing strategy.

#### 4.2.3. Controlled Sink Mobility

Controlled sink mobility is another method for lifetime enhancement which is achieved from network feedbacks. The mechanism proposed by Basagni *et al.* [10], for example, controls the multiple mobile sinks simultaneously by using a distributed heuristic approach applied in WSNs. At network set up, the entire field will be partitioned into several sites. After that, each sink node starts the training phase by flooding a request packet in which it asks all sensor nodes within the site to send back a test packet. It includes the information about the path between the sender node and the sink at corresponding site. Finally, they should share the collected information among themselves. By investigating the test packets, a sink node can estimate the incoming traffic to each relay node *q* when the source node *p* is generating data packets and sending them to the sink. On the other hand, the sensor nodes can also compute the distance from the sink and encapsulate this information in the test packet during the training phase. At the end of this phase, each trained sink selects a particular site and announces its current position to all sensor nodes. In this way, the nodes can set up routes towards the sink. During data transmission, the nodes are responsible for reporting their residual energy to the sink. The data packets carry the information about nodes' power level to the closest sink by employing a piggyback mechanism.

A sink is also able to calculate the data flow rate received from each sensor node and share the mentioned information with the other mobile sinks. Each sink periodically decides whether to relocate or not. In order to de-synchronize sink decisions, it waits for a random time before any movement. After this period of time, the sink calculates the expected lifetime improvement through relocating to an unoccupied site according to the available information about the condition of the network. If the sink node recognizes that repositioning to a new site can enhance the network lifetime more than a specific threshold, it informs the current source nodes that it is on the move and then shuts down the transmission. Then it selects the site, repositioning to which causes the maximum lifetime and informs the other sinks of its relocation decision. After that, it starts to move to the new site if the other sinks are static at the moment. As soon as it reaches the new site, it activates itself by sending a routing packet to the nearby sensor nodes in order to encourage them to construct new routes. The authors also proposed a stochastic mobility pattern in which the decision for site selection is completely random.

One of the benefits of this algorithm is that the sensor nodes have at least one active sink as a destination for their packets at any time. Since the source nodes do not need to buffer packets during sink movement, the end-to-end delay will be decreased remarkably. However, the sensor nodes have to consume additional energy for receiving availability and unavailability message which are broadcasted by sink nodes [10]. The simulation results indicate that this distributed heuristic can significantly increase the lifetime, with a gain of +77.7% in comparison with random sink mobility and on the order of +382.4% compared to a static sink approach [10].

***Mobile Sink Based Routing Protocol (MSRP)*** [11], which was proposed by Nazir and Hasbullah, is an energy-efficient hierarchical protocol that prolongs the network lifetime by employing a controlled sink mobility mechanism. In this algorithm, the sensed data will be collected from the cluster heads (CHs) by a single mobile sink which moves in the vicinity of CHs. The information about the residual energy of the CHs is also collected by mobile sink during data gathering. Based on this information, the mobile sink moves to the CHs that have higher energy levels. The data forwarding technique which is used in MSRP to send packets towards the mobile sink works as follows.

The algorithm can be divided into two main phases based on protocol operation: Setup Phase and Steady Phase. (1) *Setup Phase:* the sink node begins this phase by sending a beacon message to the neighboring nodes. After that, the sensor nodes which receive that beacon generate and forward a registration message to the sink node. Setup phase consists of three stages that are explained as follows: (A) *Initialization:* In this stage, the network will be partitioned into several clusters and cluster members send the sensed data to the corresponding CHs. Eventually, the CH nodes wait for the mobile sink to pass nearby in order to forward aggregated data to it. (B) *Mobile Sink Advertisement:* when a mobile sink reaches a predefined destination, it starts broadcasting a beacon message including its current location information. As soon as a CH node receives this message, it realizes that the mobile sink is in its vicinity and it's time to relay data packets. (C) *CH Registration:* Upon receiving a beacon message as described in the previous stage, the CH node checks whether it has previously forwarded any data packets to the mobile sink in the current cycle or not. If it has done so before, the CH simply ignores the advertisement message. Otherwise, it replies by sending a registration message back to the mobile sink and waits for an acknowledgment packet. (2) *Steady Phase:* in this phase, data gathering which is the main responsibility of the mobile sink is discussed completely. Just like the setup phase, it is also divided into three stages as follows: (A) *TDMA Scheduling:* The registered CHs should receive a TDMA-based scheduling table from the mobile sink. The table consists of time slots in which each neighboring CH node should relay its data to the mobile sink without any conflict with other CHs. (B) *Forwarding to Sink:* a multi-hop or single hop mechanism will be used by each CH to send sensed data accompanied with the CH's residual energy to the sink. The energy level of CHs can help the mobile sink make a decision for its next movement. (C) *Sink Movement:* At the first cycle, the sink trajectory is predefined, but in subsequent cycles, it relocates to the position of CHs having a higher power level in order to balance energy consumption all over the network [11].

According to the experimental results [11], the average energy per packet is decreased on the order of about 50% and 10% in comparison with static sink and multiple sink approaches, respectively. However, according to the principles of this algorithm, additional energy consumption would be imposed on sensor nodes for relaying control packets in a multi-hop manner such as periodic CH selection, advertisement broadcasting in each cycle, CHs registration messages and the messages including TDMA-based scheduling table.

### 4.3. Multi-Path Mechanism

Depending on the methods used for finding the path, routing protocols in WSNs can be classified into three groups as follows: *proactive (table-driven), reactive (on-demand*) and *hybrid*. According to the on-demand method, the route should be built only when a node decides to send data to a destination, in contrast with the table driven technique in which all of the nodes must exchange route messages periodically to maintain a permanent route table all over the network. Therefore, only the active path, in which a link failure has taken place, should be updated. A hybrid routing protocol is a combination of the both mechanisms [40]. The most popular multi-path routing protocols in wireless sensor and *ad hoc* networks are based on traditional on-demand single-path routing schemes such as *ad hoc* On-demand Distance Vector (AODV) and Dynamic Source Routing (DSR) [16].

There are two techniques for constructing multiple paths in WSNs. In *disjoint multipath* methods all of the paths are apart from each other so that there is no link/node sharing in link-disjoint or node-disjoint mechanisms, respectively. In this manner, a failure in one path cannot affect the alternate paths. In contrast with the link-disjoint scheme, node-disjoint multi-path protocols avoid sharing any nodes between the multiple routes. This means that these kinds of algorithms try to transmit the data on multiple independent paths simultaneously without any congestion on a specific node along the routes. Another method is *braided (meshed) multipath* in which the paths are partially disjoint or not disjoint at all [40].

#### 4.3.1. Braided/Meshed Multipath

**Figure 5.**
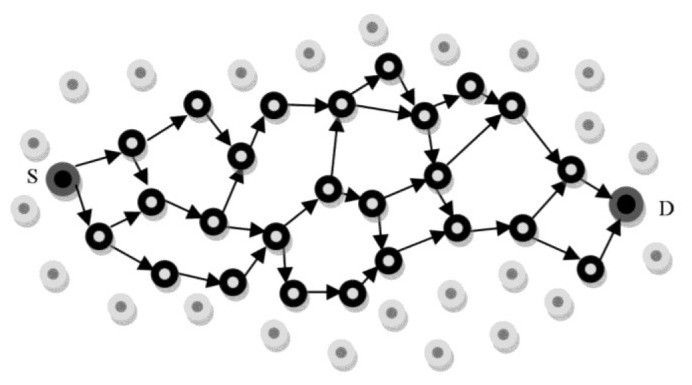
A source-to-destination meshed multipath [41].

One of the protocols that uses position information of the nodes to make braided multi-path and location-based routing is the ***Mesh Multi-Path Routing (M-MPR)*** algorithm [41] with two functional modes. The first one is M-MPR with Selective Forwarding (*M-MPR-SF*) and the other one is M-MPR with Packet Replication (*M-MPR-PR*). The M-MPR protocol is designed based on multi-path searching and multi-path routing phases. In the first phase, each node firstly broadcasts some information such as its ID, location information and residual energy to the neighboring nodes. Next step is *Route discovery* in which each of the sensor nodes tries to implement a meshed multipath towards the sink. As shown in Figure 5, an intermediate node is only allowed to accept a maximum of *two* copies of a discovery packet in order to reduce the power consumption and the receiver complexity. Meanwhile, a maximum of *two* downstream neighbors will be chosen by the intermediate node to forward the *first* arrival copy of discovery packet. This can minimize the control overhead for alternate routes towards the sink.

*Route reply* is the last step of the multi-path searching phase. As soon as the sink receives the discovery packets from its neighbors, it chooses the first two neighboring nodes and forwards the route reply messages on the links in the reverse direction. Each intermediate node relays this message until it reaches the source node. Now, the meshed multipath routes are ready to carry the data packets. In the multipath routing phase, the protocol can use either selective forwarding (SF) or packet replication (PR). In SF mode, the source should analyze each packet individually and send it through a high quality link while in PR the source follows a method based on dissemination several copies of the same packet sent on different paths simultaneously. Furthermore, both the SF and PR algorithms employ an end-to-end forward error correction (FEC) coding for successful message routing by avoiding acknowledgment-based retransmission.

Although RP could increase the throughput, it imposes a large amount of overhead on the network, especially when it uses the FEC mechanism [41]. Comparatively, energy consumption in M-MPR-PR is about 2.8 times more than in M-MPR-SF. This excessive energy wastage is because of forwarding redundant packets on multiple paths [41].

#### 4.3.2. Disjoint Multipath

##### Link-Disjoint Multipath

***Adaptive Greedy-Compass Energy-Aware Multi-path (AGEM)*** [14], for example, is a kind of link-disjoint multi-path geographic routing that uses the adaptive compass mechanism in order to select the best neighbor in the forwarding phase. The main objective of this algorithm is to maximize the network lifetime and achieve better quality of service for audio and video stream transmission in high density wireless sensors networks.

In this way, AGEM tries to distribute traffic loads on multiple paths based on a greedy routing method. The protocol has two modes as follows: the smart greedy forwarding and the walking back forwarding. According to the greedy mechanism, the first mode will be used when there is at least one neighbor closer to the sink than the sender node. On the other hand, the second mode will be used when the sender hits a void area in which none of the neighbors are eligible to be chosen based on greedy algorithm.

In smart greedy forwarding mode, each node should keep a table of its *one*-hop neighbors including some information such as the data-rate of the link, the distance of the neighbor to the sink, the estimated distance to its neighbors, and the remaining energy. Since the AGEM is proposed for mobile environment, beacon messages should be employed to update this information. An objective function “*f(x)*” is calculated as a score for each neighbor depending on the mentioned information exchanged at fixed intervals. Whereas the protocol selects the best neighbors based on the adaptive compass policy, it selects a series of nodes with smallest angular offset from a supposed line between the sender node and the destination by satisfying minimum candidate nodes to implement online multipath routing. Then among these candidates, the neighbor has the chance to be chosen which serve better the function *f(x)*. The following equation shows the elements of this function:

(6) $$f(N_{i})=N_{i\;\text{Energy}}-E_{TX}(N_{i\;\text{Distance}})-E_{RX}$$

where *E_TX_ (N_i Distance_*) = the energy needed to send a packet to the neighbor *N_i_*, and *E_RX_* = the energy needed to receive the packet. In order to meet load balancing requirements in the network, each relay node ranks the received data packets based on the number of hops they paced to reach this node. After that it forwards a data packet with the biggest number of hops towards the best neighbor while it sends a packet with the smallest number of hops towards the worst neighboring node. Meanwhile, the protocol can switch to the second mode and use walking back forwarding strategy to avoid holes. In this state, the node delegates the packet forwarding duty to the previous hop. This operation will be repeated recursively till a relay node could be reached to forward the packet successfully [14].

Experimental results [14] confirm that AGEM improves the distribution of the remaining energy across the network by more than 32% compared to the GPSR routing algorithm. The reason is that most of the nodes can be active due to multipath routing. It also significantly reduces the end-to-end delay and packet loss at a rate of 150% and 900%, respectively. It is because of the use of multiple paths for data packet transmission. Since the protocol is designed for wireless mobile sensor networks (WMSNs), a huge amount of beacon messages for topology update wastes unnecessary power across the sensor field.

##### Node-Disjoint Multipath

**Figure 6.**
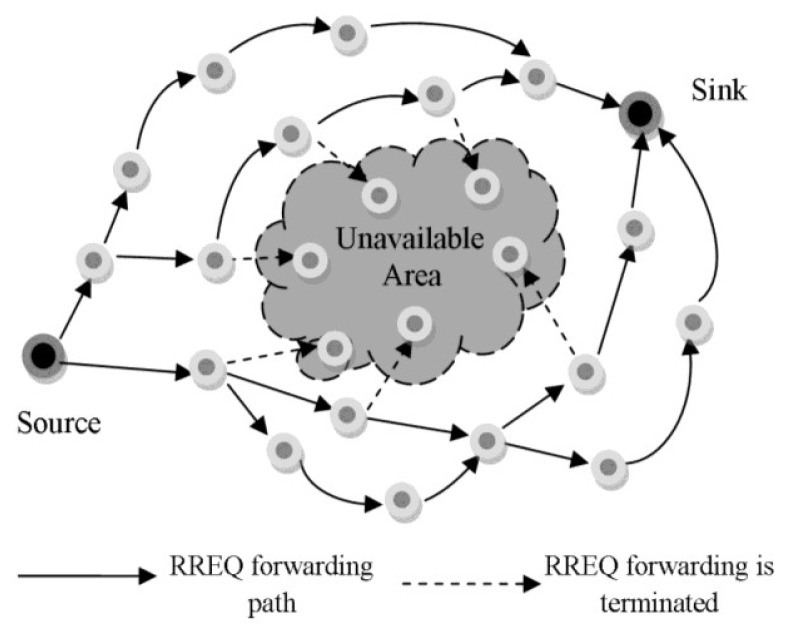
RREQ dissemination procedure in MSMRP [42].

***Micro Sensor Multi-Path Routing Protocol (MSMRP)*** [42] is an energy-aware and node-disjoint multi-path algorithm that can extend the network lifetime by distributing traffic load all over the field. It can move around the unavailable areas at the time of route construction. These areas caused by node unavailability due to energy depletion, non-uniform coverage, disasters, or extreme congestion. The protocol consists of the following procedures: (1) *Route Discovery Procedure:* as shown in Figure 6, the source node starts to broadcast a RREQ message when it has some data for transmission. The RREQ will be discarded directly, if it is forwarded to an unavailable node. (2) *Route Reply Procedure:* the sink node may receive several RREQ messages, but it chooses only two optimal paths among them and sends RREP messages back to the source node. In some circumstances, an unavailable node may appear along the inverse path. In this state, the previous hop tries to select another neighbor node specified from the cached RREP messages one by one. If it succeeds in finding one, it sends an ACK to the sink and then forwards the RREP to the source node along a new inverse path. In failure state when there is no route to the source, it sends a No message to the sink and then from the remaining paths, the sink will select another best path to send out a RREP again. (3) Route Maintenance and Error Handling Process: MSMRP uses a HELLO mechanism in the RERR process to specify a route failure. In this method, when a node does not receive HELLO message from one or several neighbors during a period, it realizes that the route through these neighbors is expired. Therefore, it prepares a RERR message and sends to their precursor node separately. It also deletes unreachable entries in its routing table. Recursively, the precursor nodes do the same process until all of the relay nodes on the route will be aware about the route expiration. To apply energy-efficiency to the proposed scheme, the protocol refuses to send RERR when it detects another unavailable node on the route [42].

Sometimes a node receives more than one RREP message from one of the precursor nodes. To ensure node disjointness among multiple paths, it sends out its neighboring table to that precursor node. After that, the precursor node searches for a common neighbor node that exists in the received table and its own table. When the precursor node chooses one common neighbor node, it sends a RREP message to it. If there is a route to the destination node of the RREP message, the common neighbor node sends an ACK message back to the precursor node. Otherwise, the precursor node selects another common neighbor node. At the worst case when there is no common neighbor, the joint node should forward the RREP by itself and the approach converts to link-disjoint multi-path method [42].

The test-bed experiments [42] show that the MSMRP algorithm could successfully build the routes crossing around the unavailable areas. However, there is no comparison with other similar protocols to justify the proposed method. Another drawback of the MSMRP protocol is that in comparison with its predecessor MSRP, it has more routing control packets such as RREQ, RREP, RERR, advertisement message of neighbor node table, HELLO message and delete message of neighbor node. These control packets impose a high overhead to the sensor nodes that cause unnecessary energy consumption across the network.

***Energy-Balance Multipath Rumor Routing (EBMRR)*** [43] is another energy-efficient approach that uses a probabilistic manner to find multiple paths between the source and the sink. In order to prolong network lifetime, it uses the available paths alternatively to distribute the energy consumption all over the network. In contrast with the original Rumor Routing [31], EBMRR considers residual energy and power usage level at the neighboring nodes. Two kinds of query messages called Forward Agent (FA) and Backward Agent (BA) are employed in this protocol. FA which is sent by source node is responsible to search for multiple paths. BA carries the information of paths and updates the routing table of relay nodes as it moves on reverse path.

The algorithm has three phases as follows: (1) *Initialization Phase:* in this phase, a HELLO message will be broadcasted by sink node to its neighbors. It consists of four fields as follows: *Type* field that shows the initialization message, *SenderID* related to the previous hop, *HopCount* that indicates the number of hops from the sink to the current receiver node, and *residual energy* of the previous hop. The HopCount and Residual energy fields are initialized by the sink node with the values 0 and ∞ respectively. When a sensor node receives a HELLO message, it checks whether the same message is registered in the memory or not. If it is received for the first time or there is a message with the same SenderID and greater Hopcount than the received one, the information of neighbors in memory should be updated. At the end of this phase, each node has sufficient information about its neighbors and the sink. (2) *Paths search phase:* when the source node has some data for transmission, it send some FA messages to find multiple disjoint paths. FA chooses the next hop node based on the following probability:

(7) $$p(j)=\{\left(\frac{E_{j}}{\sum_{k=1}^{l}E_{k}}\right)*\frac{\sum_{k=1}^{l}E_{k\_\text{toSink}}}{E_{j\_\text{toSink}}}$$

where *E_j_* = residual energy of node j and *E_k_toSink_* = E * HopCount. This parameter shows the energy consumption between the sink and node *K*. The FA message is forwarded to the next hop according to the Equation (7) until its TTL expires. As soon as the sink node receives a FA message, it generates a BA message and sends it back to the source node through the reverse path. In order to conserve more energy, the nodes that are not participating in this phase will go into sleep mode. (3) *Data Transmission and Paths Maintenance Phase:* after the source node receives all of the BA messages; it starts to send data packets to the sink by assigning a specific probability to each path. If the sink node realizes that the number of active paths is lower than two, it sends RST message to the source node and asks it to repeat the paths search phase.

In comparison to the four other protocols, simulation results [43] show a significant improvement in network lifetime. For example, the EBMRR protocol has on average 110% longer lifetime compared to its predecessor the rumor routing [31] (RR) protocol. The philosophy behind this improvement is that EBMRR distributes energy consumption all over the network by constructing multiple paths using residual energy and power usage level at the neighboring nodes. Nevertheless, it has two disadvantages. The first one is that FA and BA messages that produced by source and sink nodes, respectively, may cause collisions among the paths of different sources. The second one is that service rate and buffer capacity of the active nodes are not taken into consideration for adjusting the traffic rate of the active routes.

The authors of ***Robust and Energy-Efficient Multi-path Routing* (*REER*)** [15] proposed a method to select the best next hop during the paths construction phase based on the residual energy, Signal-to-Noise Ratio (SNR), and node available buffer size. The main objective of this protocol is to maximize the network lifetime through the traffic distribution across node-disjoint multiple paths. The algorithm has three main phases as follows: (1) *paths discovery phase*: this phase is divided into three steps. *Initialization stage:* just like directed diffusion, the sink node start to construct a series of paths towards the source node in order to use them for data transmission. In this step, each sensor node should acquire some information about its neighbors by broadcasting HELLO messages through the network. Having this information, the nodes can compute the link cost function for their neighbors according to the following equation:

(8) $$\text{Next hop}={\text{max}}_{y\in Nx}\{\alpha E_{\text{resd},y}+\beta B_{\text{buffer},y}+\gamma I_{\text{interference},xy}\}$$

During the path discovery phase, each node utilizes this function to choose the best next hop. In Equation (8), node *y* is in the vicinity of node x and *N_x_* is a set including all of the neighbors of node x. E_resd_ is the residual energy, B_buffer_ represents the buffer size, and I_interference_ is the interference between *x* and y based on signal-to-noise ratio (SNR). Moreover, *α*, *β*, and *γ* are the appropriate weights for these three factors. *Primary path discovery stage:* now, the sink can send the route request (RREQ) message to the eligible neighbor node according to the mentioned cost function in order to construct the first path. This trend will be continued by the nodes until the source node is reached. *Alternative path discovery stage:* the alternative paths are discovered with the same method but without any node sharing among the parallel paths. In order to guarantee node disjointness among multiple paths, each node only accepts one RREQ message and rejects the other ones. (2) *Path maintenance*: in order to keep the paths and cost function factors updated, the source floods KEEPALIVE messages periodically over the multiple paths. (3) *Traffic allocation and data transmission*: in this phase, the source node can select one of the following traffic allocation methods. *Data transfer through a single path (REER-1):* in this state, the protocol chooses the best path among the available paths to relay data packets until the path-cost falls below a specific threshold. Then it can switch to the next alternative path. *Data transfer across multiple paths (REER-2):* in delay-sensitive applications, REER can split up each packet to a number of equal size segments; append XOR-based error correction codes to each of them, and send the segments to the sink across multiple paths simultaneously. This can increase the reliability of data delivery and resiliency to path failures.

Simulation results [15] have shown that REER-1 improves the energy consumption by 7% to 39% in comparison with directed diffusion. When compared to REER-2 and Directed Diffusion, the changes in network size have a little effect on the energy consumption of REER-1. The reason is that the route length does not increase as the number of nodes increases. In contrast with previous link and node disjoint protocols, the REER algorithm utilizes node available buffer size in the paths construction phase in order to define the next hop efficiently. However, the main drawback of this protocol is that the source should periodically flood KEEPALIVE messages over the multiple paths in path maintenance stage. This imposes a high overhead on the individual sensor nodes that causes unnecessary energy consumption across the network.

### 4.4. Power Control Mechanism

Since sensor nodes are power-constrained and data transmission consumes a significant amount of energy, power control mechanisms can provide an energy efficient scheme with high link quality for wireless sensor networks [19]. Although the ability to vary the transmission power is in the hands of the physical layer, it is a key tool in combination with the upper layer protocols such as MAC and routing algorithms to prolong sensors' lifetime [18]. Some power control techniques proposed for routings in WSNs are discussed as follows.

Zhou *et al.* [17] presented an adaptive transmitter power approach in order to minimize the overall energy needed for sensor-to-sink communications. Their solution is based on a *Broadcast-On-Update* (BOU) method. According to this novel model, a configuration packet is used to adjust in-network nodes' transmitter power level. This packet presents the cost, the identity and the location of the node that sends it. First of all, a configuration packet is sent by the sink with a cost value of zero. Each node that receives the packet should update its node cost parameter and rebroadcast it with the new value of node cost. Then it has to calculate its own transmitter power so that it will be able to reach the neighboring node where it recently receives the configuration packet. After computing the edge cost between this node and its neighbor, the sum of the neighboring node cost and the mentioned edge cost totally in the name of path cost should be compared with the current node cost. If it is smaller, it should be assigned to the current node as its new node cost. Otherwise, the packet is dropped. At the end, the node rebroadcast the configuration packet which includes the updated node cost and its location.

Since explosive broadcasting is the main drawback of the BOU protocol, the authors presented the *BOU-WA* scheme to overcome this problem. Based on this method, the node should wait before broadcasting according to a proportional probability in order to find a better path to the sink from the energy-efficiency point of view. The simulation results [17] indicate that when the waiting time before broadcasting is increased up to a specific threshold, the energy consumption of BOU-WA will be improved by a rate of 350% more than the BOU scheme. However, the execution cost due to the complexity of computing the waiting time is the main drawback of BOU-WA algorithm.

***Adaptive Transmission Power Control (ATPC)*** [18] is another energy-efficient algorithm which is proposed to apply in several routing schemes. In this protocol, a model is built for all neighbors by each node that describes the correlation between link quality and power transmission. In order to maintain individual link quality over time dynamically, the proposed model applies a feedback-based transmission power control scheme. The authors believe that the Received Signal Strength Indicator (RSSI) and Link Quality Indicator (LQI) [44] are two suitable parameters in order to calculate the optimal transmission power level. According to this algorithm, each node proceeds to broadcast a beacon in the initialization phase but at different transmission power levels. After that, the neighboring nodes should calculate RSSI/LQI values related to these beacons and a notification packet has to send these values back to the beaconing node. Now it can use the least square approximation technique in order to specify the optimal transmission power level. Based on the feedback mechanism, the transmission power level is adjusted in the runtime tuning phase.

Although ATPC could predict the proper transmission power level accurately and reach an acceptable link quality over time, it still suffers from the initialization phase overhead. One of the other drawbacks of ATPC is that the performance of the algorithm may negatively affected by conflicting interferences and transmissions [18]. Furthermore, this protocol has the execution cost for calculating the RSSI/LQI parameters. The simulation [18] shows that ATPC conserves significantly more transmission power than other solutions. This algorithm only uses 53.6% of the transmission energy in comparison with the maximum transmission power scheme.

Kim *et al.* [19] followed a similar concept as in previous algorithm but they tried to omit the initialization phase in order to reduce the overhead. This new algorithm, which could be applied in Directed Diffusion routing protocols, is named ***On-Demand Transmission Power Control (ODTPC)***. It has two steps as follows: in the first step, a node which has data to be sent, searches in its neighbor table to find the best transmission power level. The data packet should be sent with the maximum transmission power level if there is no optimal transmission power level to the receiver. After that, the RSSI parameter related to the data packet is measured by receiver. Then the successful communication margin achieved upon the measured RSSI is returned to the sender by an ACK packet with the approximate transmission power level. Now, the sender can calculate the estimated transmission power level based on the RSSI and the margin field. In the second step, the sender gradually proceeds to adjust the transmission power level by increasing or decreasing it based on the lower and upper threshold of RSSI value over time. With the help of an analytical model, the RSSI threshold is calculated approximately. Equation (9) presents this value where: *f* = 30 bytes (the length of packet) and P_N_ = −110 dBm (the average noise floor). These parameters are achieved from experiments.

(9) $${\text{RSSI}}_{\text{THRE}}(\text{dB})=10\log\left[-1.28\ln\left(2\left(1-0.99^{\frac{1}{8\text{f}}}\right)\right)\right]+{\text{P}}_{\text{N}}(\text{dB})$$

Since the real-data and ACK packets have been exchanged with a beaconing mechanism, this algorithm has no throughput overhead. The experiments [19] in a real test-bed environment show that ODTPC consumes less than 53.48% of the energy consumption for the initialization phase in comparison with the maximum transmission power scheme. It also improves the transmission energy consumption on the entire network by rates of 50% and 120% in comparison with the ATPC and maximum transmission power schemes, respectively. Just like the ATPC protocol, this algorithm has an execution cost for calculating the RSSI parameters.

**Figure 7.**
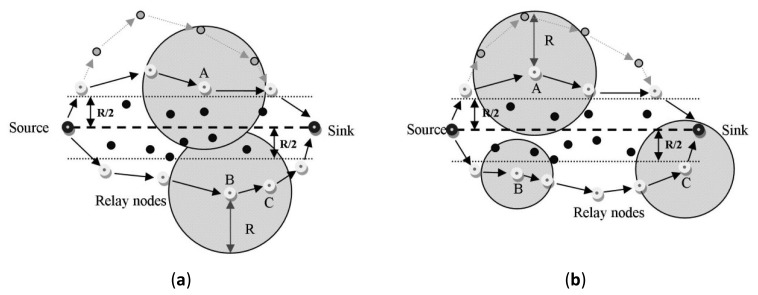
(**a**) Using maximum power level. (**b**) Power control mechanism in EECA (transmission range ≤ R).

The authors of the ***Energy Efficient and Collision Aware (EECA)*** protocol [16] also used a power control mechanism in order to save more energy in their proposal. They exploited a node-disjoint multiple path method between the source and the destination with the aid of the nodes' location information. In order to avoid the interference among two routes, the minimum distance between them should be determined to be more than R, which it is the maximum radio transmission range for each node. As shown in Figure 7, the black nodes in the area between two dotted lines are not selected by multi-path routing mechanism due to collision among the nodes located on two paths. In the route construction phase, each relay node chooses the next hop based on the three following factors: residual energy, distance to the source-sink line and progress length. These factors are employed by each next-hop candidate to calculate a back-off timer. The node having minimum back-off time wins the contest and introduces itself as the next hop.

With the help of the power control component of the protocol, it is possible to find two collision-free routes and transmit the data with minimum power in order to save the energy. The strategy of data transmission on each path is like the ODTPC algorithm. Figure 7(a) indicates the state in which node B uses the maximum transmission power level to reach the next hop C. This method wastes energy since it is not able to decrease the transmission power level to send data to node C. The usage of power control mechanism in EECA is shown in Figure 7(b), where nodes B and C use sufficient transmission power level to reach the next hop and conserve much more energy.

Through simulation [16], the authors show that EECA has 1.5% to 3% less packet loss than AODV. It also improves residual energy by around 40% to 90% in comparison with the AODV protocol. The reasons behind this improvement are that first, EECA utilizes the minimum energy needed to reach the next hop for data transmission. Secondly, the RREQ procedure is limited in the route discovery phase, which could consume unnecessary energy across the network. However, distance-based approaches for computing the interference range of the sensor nodes may fail to estimate an accurate interference [45].

### 4.5. Bio-Inspired Mechanisms

In recent years, new communications and calculation models have been originated from insect sensory systems that have directed to considerable progress like bio-inspired routing. In Ant Colony Optimization (ACO) a colony of artificial ants is one of the most biological ways to create solutions guided by information obtained from trial and error and the pheromone paths. In spite of not having much intelligence or strength, ants can successfully make colonies that consist of a very highly-organized society [22]. Some protocols based on ACO mechanism are as follows:

***Artificial Fish Swarm Optimization (AFSO)*** as a biological algorithm copying animal cooperation is the main motivation for [20] in order to suggest a new hierarchical routing protocol for WSNs. Based on the behavior of fish, this mechanism is used to solve the NP-hard problem considered to find *k* optimal clusters among the network. A central control which is implemented by the sink is used for this algorithm due to the scattering the cluster heads through the network. The sink operates this scheme at the artificial fish swarm optimization cluster head (AFSOCH) setup phase with the help of GPS. In this protocol, two other energy efficient strategies are used as follows: firstly, the sensor nodes which are not involved in data transmission phase can go to the sleep mode and conserve their energy. Secondly, by using another CH, a cluster head is able to forward data packets when it is far away from the sink node. On the other hand, a multi-hop pattern is followed by the algorithm to relay data towards the sink. Moreover, different Code Division Multiple Access (CDMA) codes can be used among the multiple clusters to have parallel communication.

Experimental results [20] demonstrate that AFSO could decrease the total energy dissipation of the network more than 20% compared to the LEACH [46] protocol. Just like its predecessor LEACH, AFSO could decrease intra-cluster and inter-cluster interferences by using two kinds of MAC protocol, CDMA and Time-Division Multiple Access (TDMA). Employing these mechanisms results in more energy conservation and network lifetime extension. However the execution overhead caused by periodic CH selection imposes unwanted energy consumption throughout the sensor field.

The ***Energy-Efficient Ant Based Routing Algorithm (EEABR)*** [47] is a new communication scheme for WSNs, which is based on the Ant Colony Optimization (ACO) metaheuristic. A colony of artificial ants that travels through the WSN is used in EEABR so that they can search for paths between the sensor nodes and a sink. To increase the lifetime of the WSN, these paths should be energy-efficient and short in length at the same time. The next hop for each ant will be selected with a probability that is a function of following factor: the amount of pheromone trail present on the connections between the nodes and the node energy. An ant travels back through the built path when it arrives at the destination node. At the same time, it updates the pheromone trail by an amount that is based on the number of nodes of the path and the energy quality. The EEABR protocol can build an energy optimized routing tree after some reiterations.

Simulation results [47] indicate that EEABR improves the average (mean) residual energy compared to the basic ant-based routing algorithm (BABR). The difference between the average values varied between 17% and 25%. Nevertheless, one of the drawbacks of EEABR scheme is that in contrast with previous algorithm, it does not consider energy conservation mechanisms based on the node status management [48]. These mechanisms which are implemented in MAC and physical layers allow the sensor nodes switching between sleep and wakeup modes and hence save energy.

Another energy-efficient algorithm based on Ant Colony Optimization is presented by authors of [49]. According to this protocol, the information is initially sent to different neighboring nodes by the source in the form of individual packages. This process is going on by each node till multiple paths are built eventually. Thus, a series of information about optimal paths will be received by the sink node. Some simulated ants as agents are used to obtain efficient routing while this operation is being performed. Since the sensor nodes lose their energy when they are in communication, the remaining energy of the nodes should also be taken into consideration as well as the lengths of the paths. Therefore, the average network lifetime would be increased by selecting nodes which have more energy in a routing task. Additionally, to achieve guaranteed delivery, acknowledgement signals are used in this scheme. When an acknowledgement is not received by a source node for a data package, it resends that package to a different path. To achieve lower energy consumption, shorter paths and transmitting data on them is chosen frequently [49].

One of the advantages of this algorithm is that it employs data aggregation to decrease the amount of messages to be transmitted and consequently conserves energy. However, it can negatively affect the accuracy of data due to the loss of details [50]. Another benefit of this ACO scheme is storing the identities of ants like sequence numbers in the node's memories, instead of saving them in the ants' memories. Pheromone values are also kept in the nodes' memory. Although it reduces the size of packets during transmission, the nodes' memory might be occupied by large lists of ants' identities and pheromone values. Simulation results [49] show that in comparison with the previously discussed EEABR protocol, the residual energy of the proposed ACO algorithm is grown by as much as 10%.

The ***Many-to-One-Improved Adaptive Routing (MO-IAR)*** protocol [51] for WSNs based on ant colony optimization and swarm intelligence (forward ants and backward ants) is another suggested ant- based algorithm to lessen the collisions with the help of a lightweight congestion control algorithm. To find the shortest and best route within a multi-hop manner in a WSN, this algorithm uses two strategies. Here each node knows its position and position of its destination. Each forward ant uses the ant-routing algorithm [52] to find the best next-hop neighboring node. Using probabilistic theory, the eligible node should be closer to the source and also closest to the sink. The binary exponential back off algorithm is used by the subsequent nodes for computing their channel access time. The shortest paths might merge or cross over at any intermediate node according to the convergence nature of the many-to-one routing scheme.

Experimental results [51] indicate that MO-IAR experiences about 230% and 110% lower collision and end-to-end delays, respectively than the Flooded Piggybacked Ant Routing (FP) [53] algorithm. The reason is that the protocol employs the shortest paths accompanied by a lightweight congestion control technique to avoid collisions.

The ***Online-Battery Aware Geographic Routing (OBGR)*** algorithm which was presented in [54] is another location-based routing protocol that maximizes the nodes' residual energy while it guarantees some QoS metrics such as end-to-end delay and network reliability. By calculating the remaining battery capacity of nodes and using this information in data forwarding decisions the network lifetime could be extended. Actually, a node with low battery capacity can turn off itself and stop its routing and sensing processes. In this state, it follows the battery recovery scheme in which the battery can improve its power level in its idle states. In the proposed algorithm, the authors used the Ant Colony Optimization (ACO) mechanism. They mentioned three factors related to the routing decision as follows: the power level of each node, the distance between the node and the sink, and the pheromone value of the link from the source to the destination which is presented as Equation (10), where *τ_i−j_* is the pheromone value of the sub-path between nodes *i* and *j*. It presents the strength of the link. *N_i−j_* is the number of times the algorithm use the link between nodes *i* and *j*. It initially is equal to 1 for all links and will be incremented by 1 whenever the link is used:

(10) $$\tau_{\text{i}-\text{j}}=\frac{{\text{N}}_{\text{i}-\text{j}}}{\sum_{\text{k}\in \text{Neighborhood}(\text{i})}{\text{N}}_{\text{i}-\text{k}}}$$

In fact, the decision rule in the ACO algorithms is the probability that any neighboring node *i* of the source node *S* will be selected as the next hop. If *j* is chosen node, then *P_j_* that shows this probability is given by the following equation:

(11) $${\text{P}}_{\text{j}}=\frac{{\text{d}}_{\text{s}-\text{j}}^{\text{w}1}\times {\text{c}}_{\text{j}}^{\text{w}2}\times \tau_{\text{s}-\text{j}}^{\text{w}3}}{\sum_{\text{i}\in \text{Neighborhood}(\text{s})}{\text{d}}_{\text{s}-\text{j}}^{\text{w}1}\times {\text{c}}_{\text{j}}^{\text{w}2}\times \tau_{\text{s}-\text{j}}^{\text{w}3}}$$

Based on Equation (11), the Euclidean distance between nodes *S* and *j* is shown by *d_s-j_. c_j_* is the battery capacity of node *j* which is equal to 0 when the node's battery is exhausted. Furthermore, information about the power level of nodes and preferred neighbors is exchanged on demand by request to send (RTS) and clear to send (CTS) messages. In comparison with GPSR [38], the OBGR protocol can significantly increase the network lifetime with a gain of 50% [54]. The philosophy behind this improvement is that OBGR schedules the power usage of nodes by utilizing an online-battery model and specify their recovery time in order to prolong their lifetime. Nevertheless, this protocol has two disadvantages: firstly, the RTS, CTS and RTR control messages impose some overhead. Secondly, compared to GPSR, the overall complexity of algorithm is a bit high.

## 5. Protocol Comparison

A common objective of all mechanisms surveyed in this paper is to prolong the network lifetime. In all approaches, it is assumed that sinks have unlimited energy resources while sensor nodes are energy constrained. Multiple and mobile sink strategies, multi-path strategy, power control schemes and bio-inspired mechanisms are examples of methods that can be employed in routing algorithms to increase network lifetime. The multi-sink and mobile sink mechanisms as discussed in Sections 4.1 and 4.2 respectively are compared in Table 1 based on the following criteria:

- *Multi-sink*: As mentioned before, the network lifetime could be improved by preventing network partitioning caused by fast energy depletion around the sink. Increasing the number of sinks is one of the methods to distribute the traffic load through the sensor field and balance energy consumption around the sinks. The algorithms presented in [5–7], are samples of multi-sink mechanisms for lifetime enhancement. Although some other protocols [8–10] are originally designed for mobile sink strategy, they can also support multi-sink mechanisms as well as previous approaches. Therefore, the researchers can use these two techniques simultaneously to get better results.
- *Mobile sink*: It is another solution for “sink neighborhood problem” caused by network partitioning around the sink. A mobile sink can replace its neighbors with low residual energy by relocating to fresh part of the network periodically. Some of the protocols [8–11] in Table 1 use this mechanism to prolong the network lifetime.
- *Multi-path*: Since employing a single path for data transmission between a source and the sink can decrease the energy level of sensor nodes on the path quickly and cause network partitioning along the route, making use of the multi-path mechanism results in traffic load and energy balancing over the sensor field. In MSDD [5] and MSLBR [7], for instance, each source node can implement multiple paths towards the multiple sinks to increase reliability and fault tolerance as far as possible. It is worth mentioning that there is no protocol listed in Table 1 using mobile sink and multi-path mechanisms simultaneously.

- *Power control*: according to this mechanism, each sensor node tries to compute the energy needed to send a packet to the next hop in multi-hop routing protocols. In this way, the node is capable to adjust the transmission power level based on the distance to the next hop and avoid using maximum power level. As a result, the network lifetime will be improved by saving nodes' energy individually on the path. Only one protocol [11] in this table can employ a power control scheme.
- *Sensor mobility*: As mentioned before, the ability to change the position of sensor nodes helps to maintain connectivity by avoiding network partitioning and sink neighborhood problems. In Table 1, none of the protocols use this method.
- *Sink movement pattern*: There are three methods used by the sink node to identify the next position during the movement. In stochastic mobility pattern [8,10], a random path is followed by the sink node while the path is predefined in a fixed [9] strategy. In controlled mobility pattern [10,11], the sink is able to define the next position autonomously based on variations of the energy factors in the sensor field. The algorithm proposed in [11] uses a fixed sink mobility method for the first round. However, it switches to controlled sink mobility in subsequent rounds.
- *Location awareness*: Location information is a powerful tool to find the best next hop in routing mechanisms. It can also be used for determining the next location of mobile nodes in the network. This information can be acquired from GPS directly or calculated on other localization methods. None of the multi-sink approaches in Table 1 are location aware. Although the sink node in all mobile sink mechanisms knows its position, there is only one [9] algorithm in which all nodes are location aware.
- *Number of sinks*: The network lifetime can be improved by increasing the number of sinks up to a specific point. When the number of sinks exceeds that point, the network lifetime is constant. The reason behind this phenomenon is that each sink becomes at most 1-hop away from a sensor node. All multi-sink approaches in Table 1 support more than one sink through the network while in some mobile sink algorithms [8–10], one or more sinks can be deployed in sensor field.
- *Network structure*: Routing algorithms in WSNs are usually classified into three group as follows: Flat (data-centric), hierarchical and geographic (location-based). Most of the protocols in Table 1 are flat-based and only one protocol [11] use hierarchical structure accompany with mobile sink mechanism to prolong network lifetime.
- *Data aggregation*: This technique can enhance the network lifetime by reducing the number of data packets transmitted in the network. Data aggregation mostly is employed in hierarchical protocols [11] where the cluster heads proceed to gather data from cluster members before they act to send them to the sink node.
- *Application Type*: This factor shows that which kind of mechanisms will be employed to send data to the sink. In time-driven method, the data are sent to the sink continuously by all or special groups of sensor nodes that caused fast energy depletion through the network. In event-driven strategy [6], on the other hand, only the data about an interested event will be forwarded to the sink while in the query-based method [5], the data should be transmitted according to the sink's request. Most of the algorithms [8–10] that support sink mobility are used for time-driven applications.
- *Sink speed*: In mobile WSNs, the sink speed is an important factor. A sink can move from one place to another by using a constant speed [8]. Some approaches [9] use a move/stop mechanism in which, the sink node moves to a new place and stops in that position for a specific period of time in order to collect data from k-hop neighbors and after that moves to another place, and so on. Sometimes the sink speed is adaptive [11] based on the number of congested areas that should be visited for data gathering.

The multi-path protocols as discussed in Section 4.3 are compared in Table 2. These algorithms are mainly aimed at distributing traffic load through the network and enhancing the network lifetime by avoiding network partitioning. These protocols are compared together according to the following criteria:

- *Lifetime improvement mechanism*: This field shows that which kind of mechanism for lifetime improvement is used in each protocol. As shown in Table 2, all protocols only use multi-path mechanism except MSMRP [42] that employs both multi-path and power control schemes simultaneously.
- *Node or link disjoint*: Disjointness is an important property for multi-path protocols. Node or link disjoint protocols try to prevent interference between multiple paths and avoid packet retransmission caused by collision. Those algorithms [42,43] in which the node-disjoint scheme is used are congestion avoided and thus, having much better performance than link-disjoint multi-path strategies [14]. Braided protocols [41] cannot guarantee the disjointness among the multiple paths.
- *Number of paths*: This factor indicates the rate of traffic distribution through the network. Whenever this metric is increased, the possibility of network partitioning will be decreased. The number of paths in some approaches [42] is specified, however in other approaches [15,43] the number of parallel routes are increased as far as possible to improve the network lifetime.

- *Network structure:* Routing algorithms in WSNs are usually classified into three groups as follows: Flat (data-centric), hierarchical and geographic (location-based). Flat networks [15,42] employ a query-based strategy in order to decrease redundant data transmission through the network and conserve a huge amount of energy. In a hierarchical architecture, nodes with higher energy are chosen as cluster heads and aggregate data from other nodes (*i.e.*, cluster member). Both position information and the greedy forwarding techniques are used by geographic routings [14,41] to establish one or more energy-efficient paths from the source nodes to the sink.
- *Application Type*: This factor shows that which kind of mechanisms will be employed to send data to the sink. In time-driven method, the data are sent to the sink continuously by all or special groups of sensor nodes that caused fast energy depletion through the network. In event-driven strategy [14,41,43], on the other hand, only the data about an interested event will be forwarded to the sink while in the query-based method [15], the data should be transmitted according to the sink's request.
- *QoS*: The routing protocols [14] that apply quality of service criteria (QoS) to the network have to balance data quality and energy consumption. So, the network has to satisfy certain QoS factors such as energy, bandwidth, and delay when delivering data to the sink.
- *Network connectivity*: The algorithms proposed in [15,41,43] assume that the sensor nodes in the network should have a connected topology while in some others this assumption is not considered. AGEM [14] is an example that makes use of mobile sensors to transmit data packets between disconnected network areas.
- *Mobility*: In a static sensor network, the sensors which located at the sink vicinity may die quickly due to transmitting a large number of data packets from the nodes which are far away from the sink. The fast energy depletion around the sink causes the network partitioning and consequently sink isolation phenomenon. Thus, changing the position of neighbors [14] or sink itself is a smart choice to keep connectivity and enhance the network lifetime.
- *Location awareness*: Location information is a powerful tool to find the best next hop in routing mechanisms or can be used for determining the next location of mobile nodes in the network. This information can be acquired from GPS directly or calculated on other localization methods. In AGEM protocol [14], for example, each node checks the location information of its neighboring nodes in route construction phase to find the best neighbor for greedy forwarding mechanism. According to greedy method, a neighboring node having maximum progress on the virtual line between the source and the sink is the best candidate to be chosen as the next hop.

The power control mechanisms from Section 4.4 are compared in Table 3 based on the following criteria:

- *Lifetime improvement mechanism*: This field shows that which kind of mechanism for lifetime improvement is used in each protocol. All algorithms in Table 3 only use power control scheme, except EECA [16] which uses power control and multi-path mechanisms simultaneously to prolong the network lifetime.

- *Power adjustment technique*: This field indicates the manner by which, two neighboring nodes collaborate with each other to compute the minimum transmission power level needed for data forwarding. In some algorithms [17], a configuration packet is used to adjust in-network nodes' transmitter power level. The other protocols [16,18,19] try to calculate some parameters such as RSSI, LQI, and margin field and attach them to data packets or ACK messages and relay them to the corresponding neighboring node. By using these parameters the sensor nodes are capable to adjust their transmitter power level efficiently.
- *Location awareness*: Nodes' coordinates can be one of parameters used for computing the distance between two nodes and the energy needed for data transmission along that link. There are two [16,17] algorithms in which all nodes are location aware.
- *Network structure*: This field is discussed before (in Table 1). It seems the algorithm proposed in [17] can be applied in all multi-hop sensor-to-sink networks while the others are employed in flat [18] or geographic [16] routing protocols.
- *Proposed routing*: Some mechanisms [17] only focus on the power control schemes, while others provide joint power control and routing protocols [16].
- *Application Type*: The algorithms proposed in [18] and [19] are employed in time-driven application while the others could be applied in event-driven routing methods.
- *Mobility*: None of the protocols in Table 3 was proposed for mobile environments.

The bio-inspired mechanisms as discussed in Section 4.5 are compared in Table 4. These algorithms are mainly aimed to enhance the network lifetime by finding optimum path. These protocols are compared together according to the following criteria:

- *Network structure*: This field is discussed before (in Table 1). The first protocol [20] in Table 4 is hierarchical while the last one [54] use location-based structure accompany with bio-inspired mechanism to prolong network lifetime.

- *Lifetime improvement mechanism*: This field shows that which kind of mechanism for lifetime improvement is used in each protocol. As shown in Table 4, all protocols only use bio-inspired mechanism.
- *Application Type*: This field is discussed before (in Table 1). Most of the algorithms [47,49,51] are used for event-driven applications and only [51] could be applied for Time and Event-driven applications simultaneously.
- *Multi-path*: Multi-path methods can avoid network partitioning by distributing traffic loads on most of the sensor nodes through several paths. In this table only [49] use this method.
- *Data aggregation*: This field is discussed before (in Table 1). The cluster heads (CHs) in hierarchical networks [20] usually employ data aggregation through the cluster members.
- *Mobility*: None of the protocols in Table 4 proposed for mobile environments.
- *Location awareness*: This field is discussed before (in Table 1). The protocols proposed in [20], [51] and [54] are location aware.

## 6. Discussion and Open Issues

In this paper, a variety of energy-efficient mechanisms applied in routing protocols are summarized and classified into appropriate categories such as multi-sink, mobile sink, multi-path, bio-inspired and power control (as a cross-layer approach) techniques. These mechanisms which are categorized according to the protocol operation could be simultaneously employed in routing schemes for achieving more energy-efficiency [23]. However, some routing algorithms may use more than one mechanism from different classes.

Future trends in routing strategies for wireless sensor networks concentrated on different directions should all follow the common goal which is prolonging the network lifetime by avoiding the network partitioning phenomenon. Supporting multiple sinks in order to balance energy consumption across the network and prolong the lifetime of the sink neighbors is a hot topic these days. In most cases, the researchers employ mobile sinks for gathering data from source nodes all over the sensor field.

Partitioning phenomena could also be avoided by employing the multi-path technique [41–43]. Using this method, the traffic load could be distributed on more than one path. Therefore, the nodes on the paths tolerate less traffic and experience lower energy consumption. As said before, experiments [27] show that data transmission with maximum power level wastes a significant amount of nodes' energy. In order to address this problem, power control schemes [16–19] could provide an energy-efficient paradigm in which a sensor node forwards the data packets to the next hop using sufficient (minimum) power level. Bio-inspired protocols [47,49,51] are also aimed to save the node's energy through constructing the shortest path between the source and the sink nodes. Furthermore, power control and bio-inspired mechanisms provide high link quality for WSNs.

Although numerous researchers have worked on different strategies in the past years, there is a high potential to improve current methods in the future. Some of the most important open research issues are as follows:

- A large number of previous approaches in the venue of routing protocols include only one energy-efficient scheme in their related algorithms. However, a combination of different methods could be applied in new researches. For instance, implementing multiple paths towards multiple static sink with the help of bio-inspired techniques for path optimization is an open issue. On the other hand, using power control scheme accompany with multi-path mechanism helps the protocol to conserve much more energy [16]. It seems using a hybrid form of multiple and mobile sinks is a smart choice for more lifetime elongation [8]. The dual-sink [56] protocol, for example, is a novel scheme in which one of the sinks is mobile and collects the data packets from one or a few hops neighboring nodes while the other one is stationary at the center of field and receives data from far away source nodes without any localization overhead. In this way, the algorithm could benefit the advantages of both static and mobile sink approaches. Therefore, the hybrid multiple mobile sinks is an open issue for future trends.
- Cross-layer paradigms could be employed to conserve more energy, hence prolonging network lifetime. To this end, network layers can use power control mechanisms at the physical layer to restrict transmission range at the time of packet forwarding. This can reduce the energy consumed by a transmitter dramatically [16].
- One of the drawbacks of mobile sink methods is that in practice, the mobile sink may not be allowed to move along straight lines; for instance, boundaries or obstacles may block the moving paths of the sink. The solution of this problem is known as *obstacle avoidance methods* [57]. More research should be done to overcome this problem.
- Imaging and video sensors in real-time applications pose issues like Quality of Service (QoS). In QoS algorithms some metrics such as delay and bandwidth should be guaranteed during the network functionalities. Satisfying these metrics, especially in mobile sink scenarios, may be in conflict with achieving more energy-efficiency [58]. Improving sink mobility methods or using multi-path routing approaches [14] in QoS algorithms is needed to conserve much more energy.
- Most of the multi-sink approaches [5,6] suffer from control overhead caused by sinks' advertisement flooding in the gradient field construction phase. Mitigating the negative effect of such overhead on energy-efficiency is an open research issue.
- Mobile sink mechanisms are mostly used for time-driven applications in which the sink moves across the network and collects the data from CHs [11] or source nodes themselves [8–10]. However, there are a lot of applications that employ event-driven paradigms [56] for data forwarding. Therefore, mobile sink strategy should be improved for data gathering in these kinds of networks.
- The main drawback of power control schemes is the execution cost due to calculating the RSSI/LQI parameters [17–19]. Therefore, mitigating the overhead caused by such calculation could be an open issue for future trend.
- Current energy-efficient mechanisms that are proposed to prolong network lifetime do not consider security since their final goal is not satisfying this matter. For example, in multi-sink strategy [5–7], the probability that a malicious node plays the role of sink node and collects the data is very high. In this way, it can negatively manipulate the identity of data. Thus, the security issues in all mentioned mechanisms should be more taken in to consideration.
- Finally, a strategy based on joint routing protocols and energy-efficient schemes like sink mobility could be very efficient, since routing and these techniques have a close interaction on each other [9].

## 7. Conclusions

In wireless sensor networks, the nodes which are located on a non-optimal single path and forward data packets with maximum transmission power level may run out of energy quickly. This causes network partitioning along the paths through the sensor field. Furthermore, the sink neighbors tend to lose their energy much faster than the nodes which are far away from the sink due to the fact they are carrying heavier traffic loads. This also results in network partitioning around the sink and consequently causes sink isolation phenomena. All these problems can decrease the network lifetime significantly. In recent years, many approaches were proposed to address these problems. Nevertheless, there is a need to discuss and classify these methods as well as investigate their advantages and weakness points. In this paper, we present a new classification of the fundamental mechanisms that are applied in routing protocols to prolong the network lifetime. Figure 2 showed this taxonomy in detail. These mechanisms are categorized into five groups: multi-sink, mobile sink, multi-path, power control and bio-inspired schemes. Among them, power control is definitely a cross-layer technique including routing and physical features while the rest are simultaneous schemes which are applied in routing protocols. We discuss all mechanisms in detail, with an emphasis on their advantages and disadvantages as well as their significance. Comprehensive comparisons of these methodologies are presented in Tables 1–4 based on their inherent characteristics. Although these energy-efficient mechanisms look promising, there are still many challenges that need to be resolved in order to improve sensor network lifetime. We note those challenges and have highlighted future research trends in this regard.

## Figures and Tables

**Table 1.** Comparison of multi-sink and mobile sink mechanisms.

**Protocol**	**Metrics of Comparison**											

	**Multi-Sink**	**Mobile Sink**	**Multi-Path**	**Power Control**	**Sensor Mobility**	**Sink Movement Pattern**	**Location Awareness**	**Number of Sinks**	**Network Structure**	**Data Aggregation**	**Application Type**	**Sink Speed**
**MSDD** [5]	Yes	No	Yes	No	No	N/A	No	K (*k* ≥ *2*)	Flat	Yes	Query-Driven	N/A
**GLOBAL** [6]	Yes	No	No	No	No	N/A	No	K (*k* ≥ *2*)	Flat	No	Event & Time-Driven	N/A
**MSLBR** [7]	Yes	No	Yes	No	No	N/A	No	K (*k* ≥ *2*)	Flat	No	Time-Driven	N/A
[8]	Yes	Yes	No	No	No	Random	Yes *Only Sinks*	K (*k* ≥ *1*)	Flat	No	Time-Driven	Const
[9]	Poss.	Yes	No	No	No	Fixed	Yes	K (*k* ≥ *1*)	Flat	No	Time-Driven	Const
[10]	Yes	Yes	No	No	No	Controlled & Random	Yes *Only Sinks*	K (*k* ≥ *1*)	Flat	No	Time-Driven	N/S
**MSRP** [11]	No	Yes	No	Poss.	No	Controlled	Yes *Only Sink*	1	Hierar-chical	Yes	N/S	Adaptive

**Table 2.** Comparison of multi-path routing protocols.

**Protocol**	**Metrics of Comparison**								

	**Lifetime Improvement Mechanism**	**Node or Link Disjoint**	**Number of paths**	**Network Structure**	**Application Type**	**QoS**	**Network Connectivity**	**Mobility**	**Location Awareness**
**M-MPR-SF** [41]	Multi-path	Braided (*Meshed*)	N/S	Location-Based	Event-Driven	No	Yes	No	Yes
**AGEM** [14]	Multi-path	Link- Disjoint	N/S	Location-Based	Event-Driven	Yes	No	Yes *Only Sensors*	Yes
**MSMRP** [42]	Multi-path & Power Control	Node- Disjoint	2	Flat	N/S	No	No	No	No
**EBMRR** [43]	Multi-path	Node- Disjoint	K (*k* ≥ *2*)	Flat	Event-Driven	No	Yes	No	No
**REER** [15]	Multi-path	Node- Disjoint	K (*k* ≥ *2*)	Flat	Query-Driven	No	Yes	No	No

**Table 3.** Comparison of power control schemes.

**Protocol**	**Metrics of Comparison**						

	**Lifetime Improvement Mechanism**	**Power Adjustment Technique**	**Location Awareness**	**Network Structure**	**Proposed Routing**	**Application Type**	**Mobility**
**BOU-WA** [17]	Power Control	By configuration packet	Yes	Multi-Hop Sensor-to- Sink Network	Not Specified	Event-driven	No
**ATPC** [18]	Power Control	Feedback-Based (RSSI/LQI)	No	Flat	RPAR [55]	Time-driven	No
**ODTPC** [19]	Power Control	Feedback-Based (RSSI/Margin Field)	No	Flat	DD [29]	Time-driven	No
**EECA** [16]	Power Control & Multi-path	Feedback-Based (RSSI/Margin Field)	Yes	Geographic	EECA [16]	Event-driven	No

**Table 4.** Comparison of bio-inspired mechanisms.

**Protocol**	**Metrics of Comparison**						

	**Network Structure**	**Lifetime Improvement Method**	**Application Type**	**Multi-path**	**Data Aggregation**	**Mobility**	**Location Awareness**
[20]	Hierarchical	Only Bio-Inspired	N/S	No	Yes	No	Yes
**EEABR** [47]	N/A	Only Bio-Inspired	Event-Driven	No	No	No	No
[49]	N/A	Only Bio-Inspired	Event-Driven	Yes	Yes	No	No
**MO-IAR** [51]	N/A	Only Bio-Inspired	Time & Event-Driven	No	No	No	Yes
**OBGR** [54]	Location-Based	Only Bio-Inspired	N/S	No	No	No	Yes
